# Synthesis and characterization of π–extended “earring” subporphyrins

**DOI:** 10.3762/bjoc.14.170

**Published:** 2018-07-30

**Authors:** Haiyan Guan, Mingbo Zhou, Bangshao Yin, Ling Xu, Jianxin Song

**Affiliations:** 1 Key Laboratory of Chemical Biology and Traditional Chinese Medicine Research (Ministry of Education of China), Key Laboratory of Application and Assemble of Organic Functional molecules, Hunan Normal University, Changsha 410081, P. R. China

**Keywords:** aromaticity, earring subporphyrin, π-extended, supramolecular chemistry

## Abstract

A π-extended “earring” subporphyrin **3** was synthesized from β,β′-diiodosubporphyrin and diboryltripyrrane via a Suzuki–Miyaura coupling and following oxidation. Its Pd complex **3Pd** was also synthesized and both of the compounds were fully characterized by ^1^H NMR, MS and X-ray single crystal diffraction. The ^1^H NMR spectra and single crystal structures revealed that aromatic ring current did not extend to the “ear” in both of the two compounds. Their UV–vis/NIR spectra were recorded and the absorption of both compounds is extended to the NIR region and that the absorption of **3Pd** is further red-shifted and more intense.

## Findings

Since its first synthesis in 2006 [1–2], subporphyrin, the lowest homolog of porphyrins, has received considerable attention [3–8] due to its 14π-electron configuration and bowl-shaped structure. In addition the intense absorption in the UV–vis region [9–24] makes it a promising building block in pigments. The functionalization of subporphyrin can proceed at various sites such as the central boron atom [25–30], *meso*- [31–32] and β-position [33–35]. By using the method developed by Osuka the β,β′-diborylsubporphyrins [36] can be obtained in high yields. A subsequent Suzuki–Miyaura coupling smoothly affords various β-aryl-substituted subporphyrins [37]. Alternatively, some β-aryl/vinyl-substituted subporphyrins can be synthesized from aryl/vinyl borate and the corresponding β,β′-dihalosubporphyrins, which can be obtained by the treatment of β,β′-diborylsubporphyrins with NBS/NIS in the presence of a Cu(I) salt [36].

Recently, our group successfully prepared multiple cavities π-extended “earring” porphyrins through the aforementioned Suzuki–Miyaura coupling reaction and subsequent oxidation [38]. In this case β,β′-dibromo/tetrabromoporphyrins and diboryltripyrrane were applied as reactants. We discovered that both the π-extended “earring” porphyrins and the corresponding Pd complexes exhibited remarkably near-infrared absorptions.

**Scheme 1:**
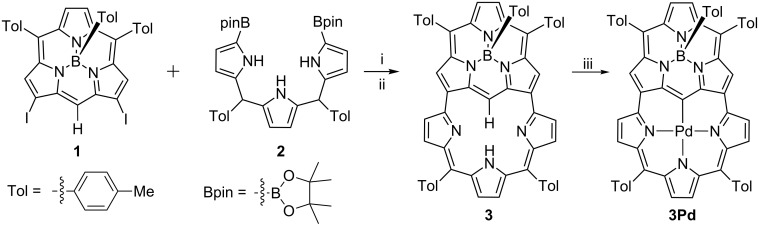
Synthesis of “earring” subporphyrin and its Pd complex. Synthetic procedure: (i) Diboryltripyrrane (2.5 equiv), Pd_2_(dba)_3_ (10 mol %), SPhos (2-dicyclohexylphosphino-2',6'-dimethoxybiphenyl, 40 mol %), Cs_2_CO_3_ (2 equiv), CsF (2 equiv), Tol/DMF 2:1, reflux, 48 h; (ii) in air, rt, overnight; (iii) Pd(OAc)_2_ (3 equiv), CH_2_Cl_2_/MeOH 5:1, rt, overnight.

Based on our previous work, we herein designed a subporphyrin with one “earring”. The different geometry and properties could be envisioned due to the bowl-shaped structure and 14π-electron configuration of subporphyrin. To construct the skeleton of the “earring” subporphyrin, we performed a Suzuki–Miyaura coupling reaction between β,β′-diiodosubporphyrin **1** [37] and diboryltripyrrane **2** [38] (Scheme 1). Monitored by TLC, we merely observed a complicated mixture without any major band during the progress of the reaction. However, several clear bands emerged after stirring the mixture overnight at ambient conditions. This observation revealed that the coupling products could be oxidized by air and thereafter the target “earring” subporphyrin **3** was obtained in an isolated yield of 15% after column chromatography.

**Figure 1:**
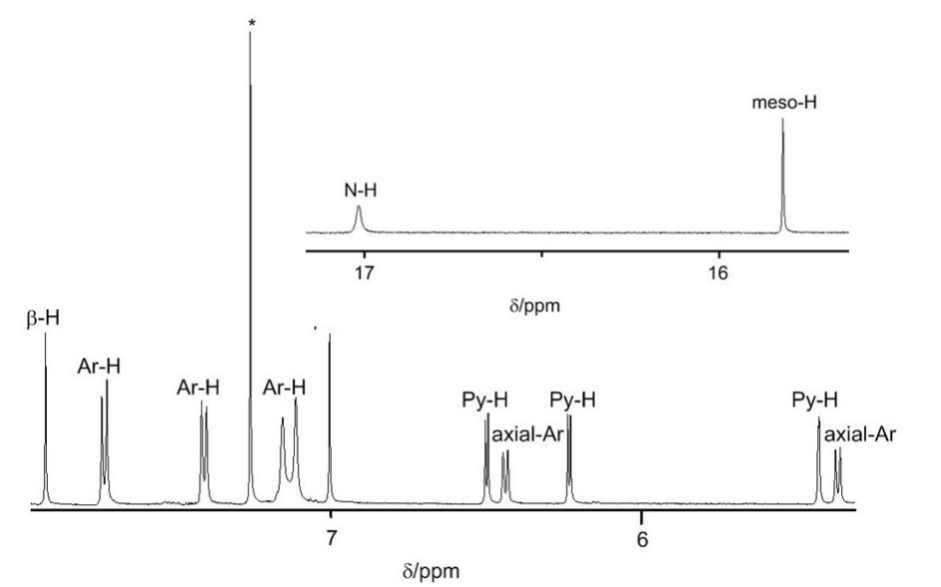
Partial ^1^H NMR spectrum of **3**.

The mass spectrum of **3** exhibits a parent-ion peak at *m*/*z* = 911.4036 (calcd for C_64_H_48_BN_6_ [M]^+^ = 911.4028), which is in agreement with its structure. The ^1^H NMR spectrum of **3** (Figure 1) indicates it a symmetric structure. The peak appearing at 17.02 ppm is assigned to the NH since a D_2_O exchange experiment lead to its disappearance. While the peak at 15.82 ppm can be assigned to the *meso*-H of the subporphyrin moiety, which is shifted to a lower field region in comparison to 8.60 ppm of 4-tolyl-(5,10-di-(4-tolyl)-subporphyrinato)boron(III) (**1a**). These two signals at 17.02 ppm and 15.82 ppm can be attributed to the antiaromatic character of the ear-containing macrocycle, which is quite similar to the analogous “earring” porphyrin [38]. Furthermore, the antiaromaticity of the ear-containing macrocycle is proved by the large positive NICS value in the hole as well as the anticlockwise ring currency (see Supporting Information File 1 for details).

**Figure 2:**
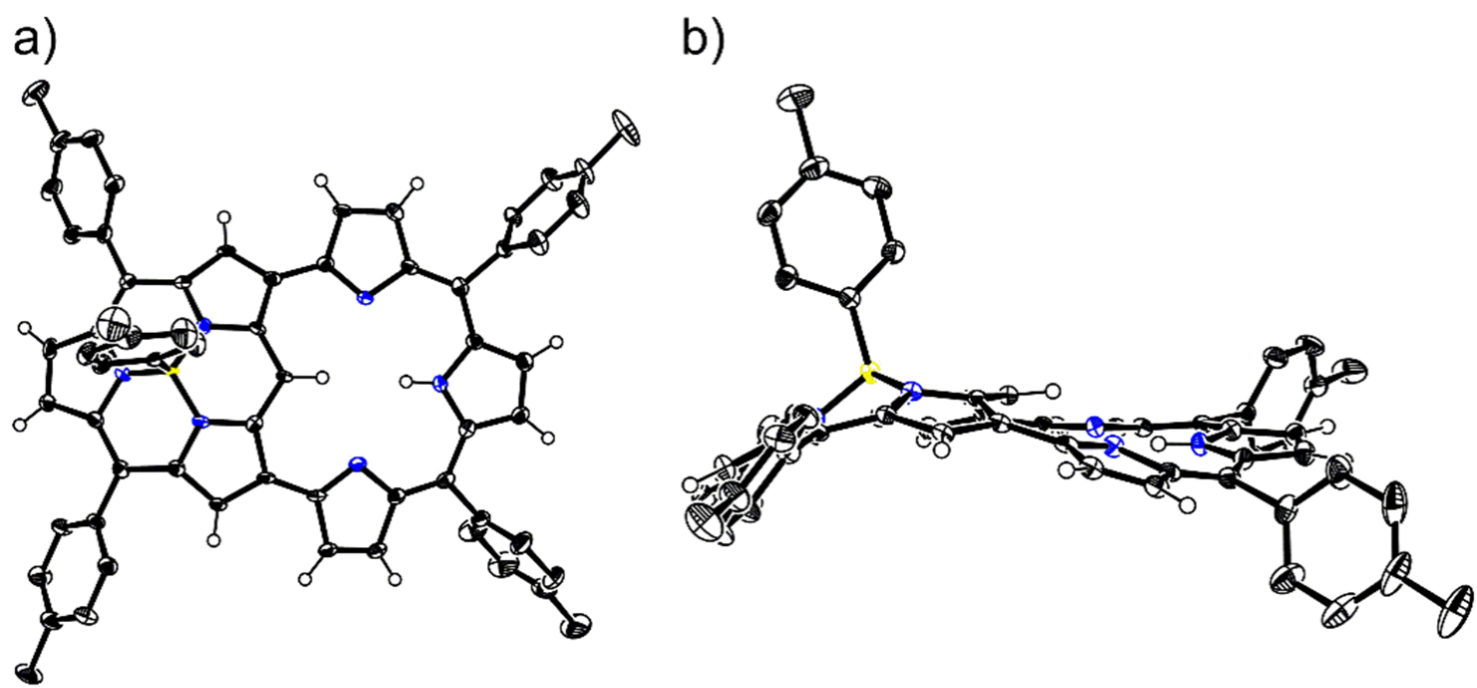
X-ray crystal structures of **3**: a) top view; b) side view. Thermal ellipsoids are drawn at the 50% probability level. All hydrogens on tolyl groups are omitted for clarity.

Although some characteristic peaks in the ^1^H NMR spectrum of the “earring” subporphyrin are quite similar to those of “earring” porphyrin, there are some differences in their structures. This is mainly due to the fact that the bowl-shaped subporphyrin core differs significantly from the saddle-shaped or nearly planar porphyrin core. To elucidate the differences in their structures, we endeavored to cultivate single crystals of **3** and collected the data. The diffraction data unambiguously confirmed the designated structure (Figure 2) and revealed that all peripheral C–C bonds in the subporphyrin moiety are of similar lengths (1.383(6)–1.447(5) Å). In contrast, the C–C bond lengths in the tripyrrin moiety alternate (1.341(5)–1.472(5) Å). These data clearly indicate that the subporphyrin moiety remains its aromaticity while the tripyrrin moiety participates in an antiaromatic system as shown in Scheme 1. The cavity surrounded by the tripyrrin moiety owns a long axis of 4.229(4) Å and a short axis of 4.201(5) Å, which are almost the same. Despite of this the cavity is not circular because the three N atoms in the tripyrrin moiety and the nearest *meso*-C of the subporphyrin moiety are not ideally coplanar. This feature can be proved by the dihedral angles of two adjacent pyrrole units in the tripyrrin moiety, which are as large as 15.8(1)° and 16.1(1)°, respectively. We assume that the twisted structure results from the strain transmitted from the subporphyrin moiety. Furthermore, we speculate that this strain should be the origin of the much lower yield of **3** comparing to the corresponding “earring” porphyrins.

Concerning the diameter of the cavity and the strain of the skeleton, we assumed that some metal ions may insert into the hole surrounded by the tripyrrin moiety. Experimentally, **3Pd** can be formed quantitatively by simply mixing Pd(OAc)_2_ with a CH_2_Cl_2_/MeOH solution of **3** at room temperature. This transformation is confirmed by MS with a parent-ion peak at *m*/*z* = 1015.24 (calcd for C_64_H_46_BN_6_Pd, [M]^+^ = 1015.29).

**Figure 3:**
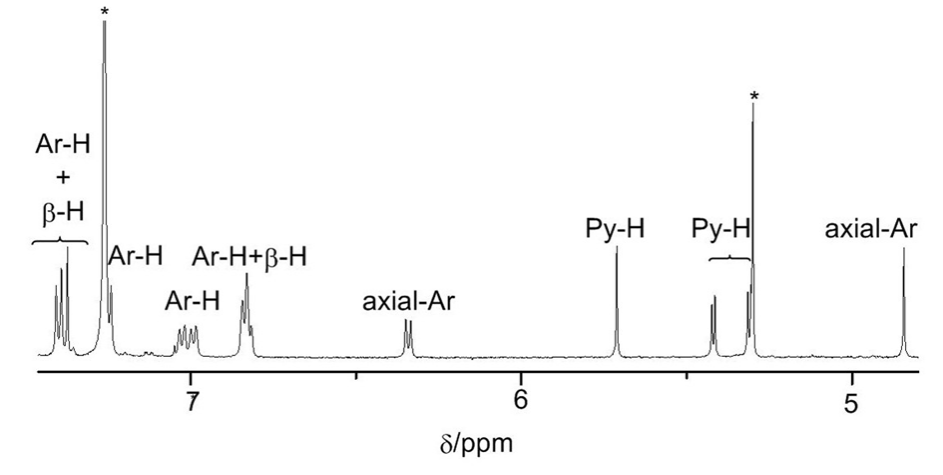
Partial ^1^H NMR spectrum of **3Pd**.

In the ^1^H NMR spectrum of **3Pd** (Figure 3), no signals are detected in the very low field region. This indicates that the Pd is inserted in the cavity of **3** with the deprotonation of both N-H and *meso*-H. Meanwhile all signals belong to aromatic hydrogens shifted to a slightly higher field region after the complexation with Pd, which reveals that the insertion of the metal does not change the antiaromatic pathways in **3**.

**Figure 4:**
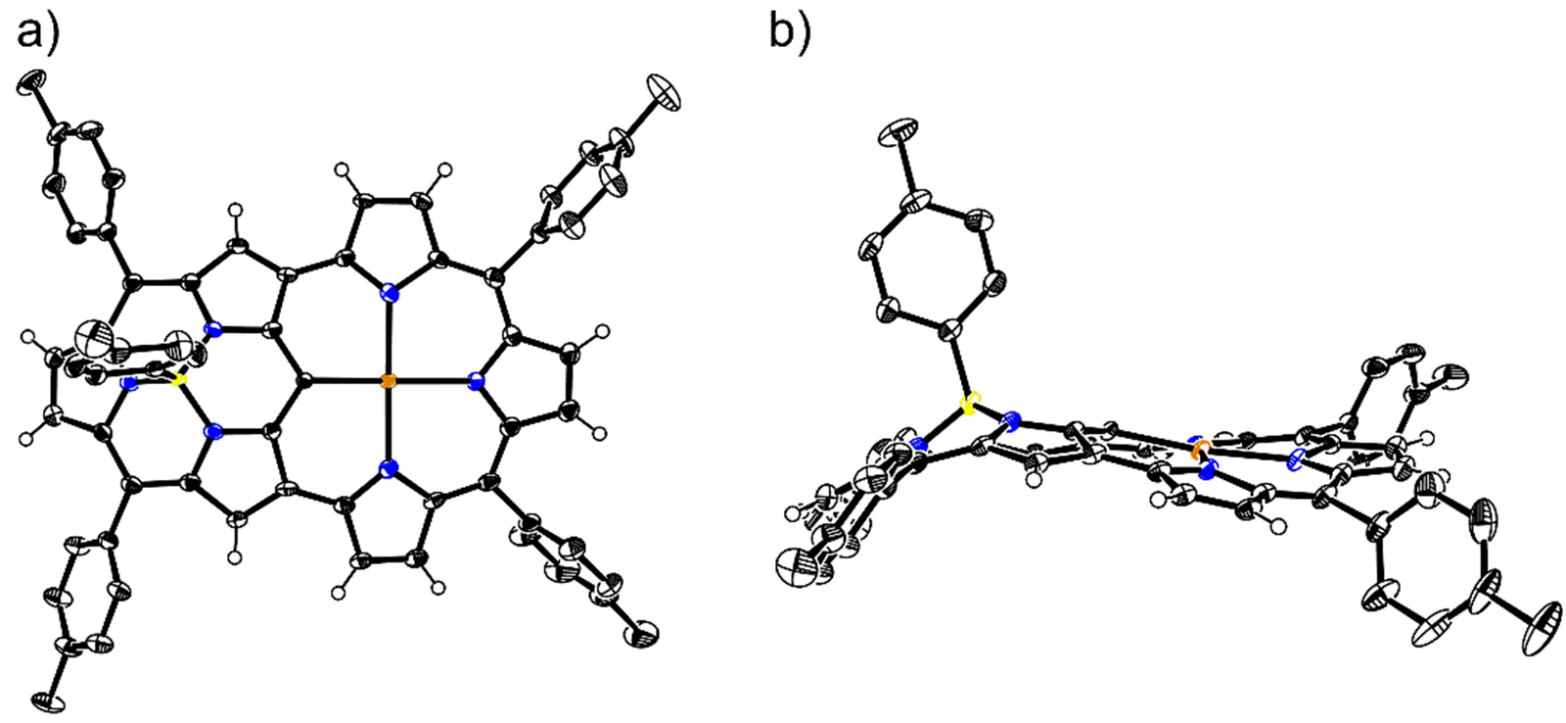
X-ray crystal structures of **3Pd**: a) top view; b) side view. Thermal ellipsoids are at the 30% probability level. All hydrogens on tolyl are omitted for clarity.

Fortunately we obtained single crystals of **3Pd** from its CH_2_Cl_2_/CDCl_3_/MeOH solution via vapor diffusion. All of the bond lengths were carefully measured based on the diffraction results (Figure 4). We found that despite all peripheral C–C bond lengths in the tripyrrin moiety were still alternating (1.348(5)–1.441(4) Å), the differences among them were somewhat smaller compared to those of **3**. While the cavity surrounded by the tripyrrin moiety in **3Pd** has a long axis of 4.123(3) Å (N–N distance) and a short axis of 4.076(4) Å (N–C distance), both of which are shorter than that of **3**. This contraction is probably due to a slight mismatch of the radii between the Pd center and the cavity. In addition, this mismatch also leads to a further twist of the pyrrole units in the tripyrrin moiety. The dihedral angles of two adjacent pyrrole units in the tripyrrin moiety are 13.7(1)° and 18.4(1)°, respectively. The difference between the two dihedral angles is much larger than that in **3**.

**Figure 5:**
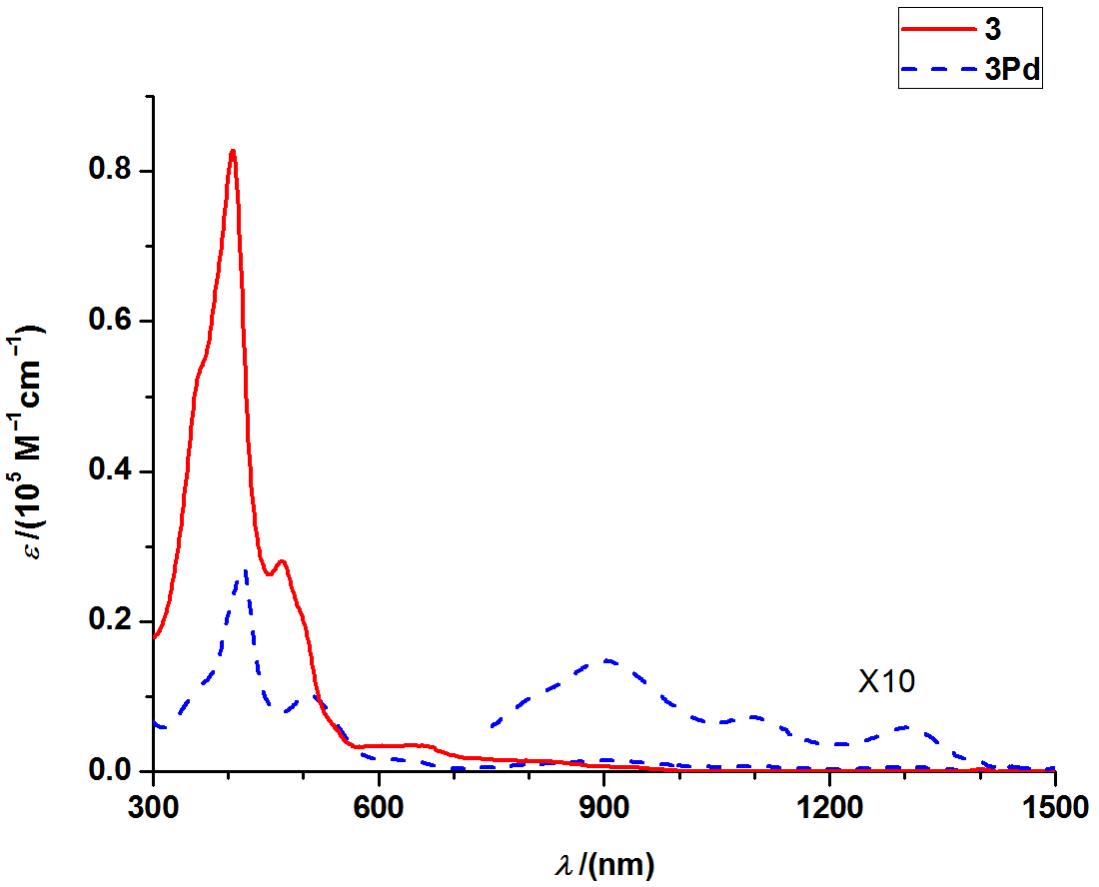
UV–vis/NIR spectra of **3** and **3Pd**.

The UV–vis/NIR absorption spectra of **3** and **3Pd** are shown in Figure 5. However, no fluorescence emission can be observed for **3** and **3Pd**. Both **3** and **3Pd** display broad Soret bands and Q-like bands and all bands are red-shifted compared to the corresponding bands of **1a**. In addition the absorptions of the Soret bands in **3** and **3Pd** are much weaker than in case of **1a**. For **3**, its tail of Q-like band extends to over 1000 nm. While for **3Pd**, its tail of Q-like band extends to over 1400 nm with several observable peaks. This remarkable absorption in the NIR region is comparable with that of the analogue “earring” porphyrins and reveals the π-conjugation between the subporphyrin and tripyrrin moiety.

## Conclusion

In summary, we synthesized a π-extended “earring” subporphyrin from β,β′-diiodosubporphyrin and diboryltripyrrane via a Suzuki–Miyaura coupling and following oxidation. This “earring” subporphyrin’s cavity allows for complexation of Pd atom to form the corresponding Pd complex. The ^1^H NMR spectra of the compounds reveal that the aromatic ring current does not extend to the “ear”. Both the structure of “earring” subporphyrin and that of its Pd complex were elucidated by X-ray single crystal diffraction analysis. In addition, their UV–vis/NIR spectra revealed that the absorption region is extended to the NIR region and that the absorption of the Pd complex is further red-shifted and more intense. This work extends the research of “earring” porphyrins to “earring” subporphyrins. Investigations on their photophysical properties and further functionalization are underway.

## Supporting Information

File 1 Experimental part.

File 2 Crystallographic information file of compound **3**.

File 3 Crystallographic information file of compound **3Pd**.
