# Supplementary material for: Synthesis and characterization of π–extended “earring” subporphyrins
Source: Beilstein J Org Chem. 2018 Jul 30;14:1956–60. doi: 10.3762/bjoc.14.170 (PMC6122274; doi:10.3762/bjoc.14.170)
Supplement: File 1 — Experimental part. [file Beilstein_J_Org_Chem-14-1956-s001.pdf]

Supporting Information
for
Synthesis and characterization of π–extended "earring" subporphyrins

Haiyan Guan, Mingbo Zhou, Bangshao Yin, Ling Xu* and Jianxin Song*

Address: Key Laboratory of Chemical Biology and Traditional Chinese Medicine Research (Ministry of Education of China), Key Laboratory of Application and Assemble of Organic Functional molecules, Hunan Normal University, Changsha 410081, P. R. China

Email: Ling Xu - xulingchem@hunnu.edu.cn; Jianxin Song - jxsong@hunnu.edu.cn

*Corresponding author

Experimental part

Table of Contents

Instrumentation and materials.. S2
General procedures S2
Compounds data.. S3
Spectra of compounds S4
X-Ray crystal data... S7
ACID diagram and NICS value data S11
References S12

Instrumentation and materials

^{1}H NMR (500 MHz) spectra were taken on a Bruker AVANCE-500 spectrometer and chemical shifts were reported as the delta scale in ppm relative to $CHCl_3$ as internal reference for ^{1}H NMR (δ = 7.260 ppm). UV–vis absorption spectra were recorded on a Shimadzu UV-3600 spectrometer. ESI-FTICR mass spectra were obtained with a Bruker Solarix XR spectrometer, MALDI-TOF/TOF mass spectra were obtained with an AB Sciex spectrometer. X-Ray data were taken on an Agilent SuperNova X-ray diffractometer equipped with a large area CCD detector. Unless otherwise noted, materials obtained from commercial suppliers were used without further purification.

General procedures

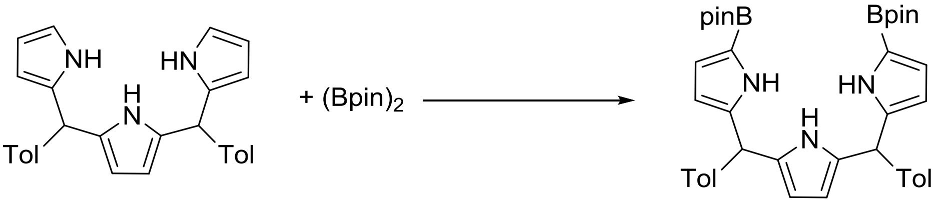

2

Synthesis of 2: A flask containing tripyrrane (284 mg, 0.7 mmol), $(Bpin)_2$ (335.6 mg, 1.4 mmol), dtbpy (5.6 mg, 0.021 mmol), and $[Ir(cod)OMe]_2$ (7.0 mg, 0.01 mmol) was purged with argon, and then charged with 1,4-dioxane (6.0 mL). The resulting mixture was stirred at reflux for 24 h. Afterwards the reaction mixture was passed through a flash silica-gel column ($CHCl_3$ as an eluent). The solvent was evaporated in vacuo to afford the product as brown solid, which was used in the next step without further purification.

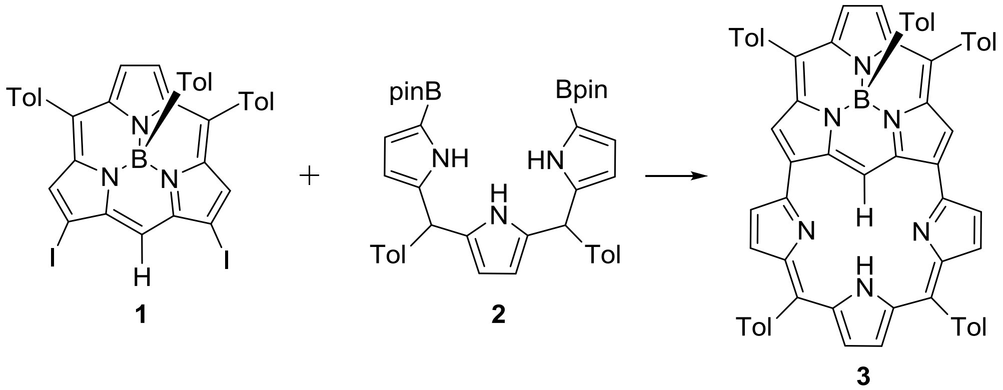

3

Synthesis of 3: A flask containing β,β′-diiodosubporphyrin **1** (50.8 mg, 0.066 mmol), diboryltripyrrane **2** (113.6 mg, 0.173 mmol), $Pd_2(dba)_3$ (6.0 mg, 0.007 mmol), Sphos (11.8 mg, 0.029 mmol), Cs_2CO_3 (50.3 mg, 0.15 mmol) and CsF (21.3 mg, 0.14 mmol) was purged with argon, and then charged with toluene (3.0 mL) and DMF (1.5 mL). The mixture was degassed through three freeze-pump-thaw cycles, and the reaction flask was purged with argon again. After refluxing for 48 h the reaction mixture was diluted with toluene, washed with water, dried over anhydrous sodium sulfate and passed through a flash silica gel column. The orange-brown solution was collected and stirred under ambient conditions overnight. Then the solvent was

evaporated in vacuo and the product purified by column chromatography (CH_2Cl_2/*n*-hexane as the eluent). Product **3** (8.9 mg, 0.0098 mmol, 15% yield) was obtained as a brown solid.

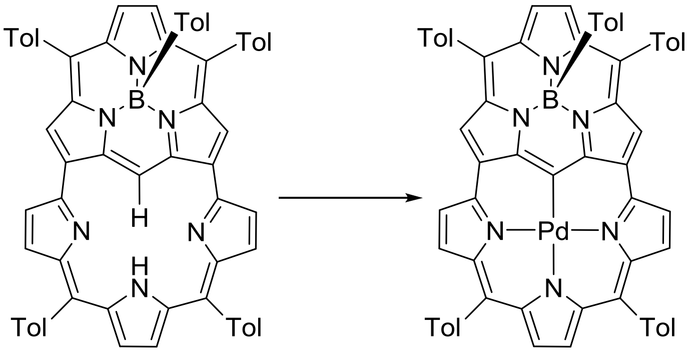

3 **3Pd**

Synthesis of 3Pd: A CH_2Cl_2/MeOH (5 mL/1 mL) solution of **3** (3.9 mg, 4.3 μmol) was placed in a round-bottomed 25 mL flask containing a magnetic stirring bar. $Pd(OAc)_2$ (2.9 mg, 12.9 μmol) and $Na(OAc)_2$ (1.3 mg, 15.8 μmol) were then added, and after being stirred overnight at room temperature, the solvent was evaporated in vacuo. The product was purified by column chromatography (CH_2Cl_2/*n*-hexane as the eluent). Product **3Pd** (4.4 mg, 4.3 μmol, > 99% yield) was obtained as a dark red-brown solid.

Compounds data

3: ^{1}H NMR (500 MHz, $CDCl_3$) δ = 17.02 (br, 1H; NH), 15.82 (s, 1H; *meso*-H), 7.92 (s, 2H; β-H), 7.73 (d, J = 8.0 Hz, 4H; Ar-H), 7.41 (d, J = 7.9 Hz, 4H; Ar-H), 7.21–7.08 (m, 8H; Ar-H), 7.00 (s, 2H; β-H), 6.50 (d, J = 4.6 Hz, 2H; Py-H), 6.44 (d, J = 7.7 Hz, 2H; axial-Ar-H), 6.23 (d, J = 4.6 Hz, 2H; Py-H), 5.43 (d, J = 1.6 Hz, 2H; Py-H), 5.37 (d, J = 7.8 Hz, 2H; axial-Ar-H), 2.51 (s, 6H; Me), 2.37 (s, 6H; Me), 1.95 (s, 3H; Me).

3Pd: ^{1}H NMR (500 MHz, $CDCl_3$) δ = 7.40 (d, J = 8.0 Hz, 2H; Ar-H), 7.37 (s, 2H; β-H), 7.24 (d, 4H; Ar-H, partial overlap with residual signal of $CDCl_3$), 7.04–6.96 (m, 4H; Ar-H), 6.87–6.80 (m, 6H; Ar-H and β-H), 6.34 (d, J = 7.6 Hz, 2H; axial-Ar-H), 5.71 (br, 2H; Py-H), 5.42 (d, J = 4.9 Hz, 2H; Py-H), 5.31 (d, J = 5.0 Hz, 2H; Py-H), 4.84 (br, 2H; axial-Ar-H), 2.41 (s, 6H; Me), 2.27 (s, 6H; Me), 2.19 (s, 3H; Me).

Spectra of compounds

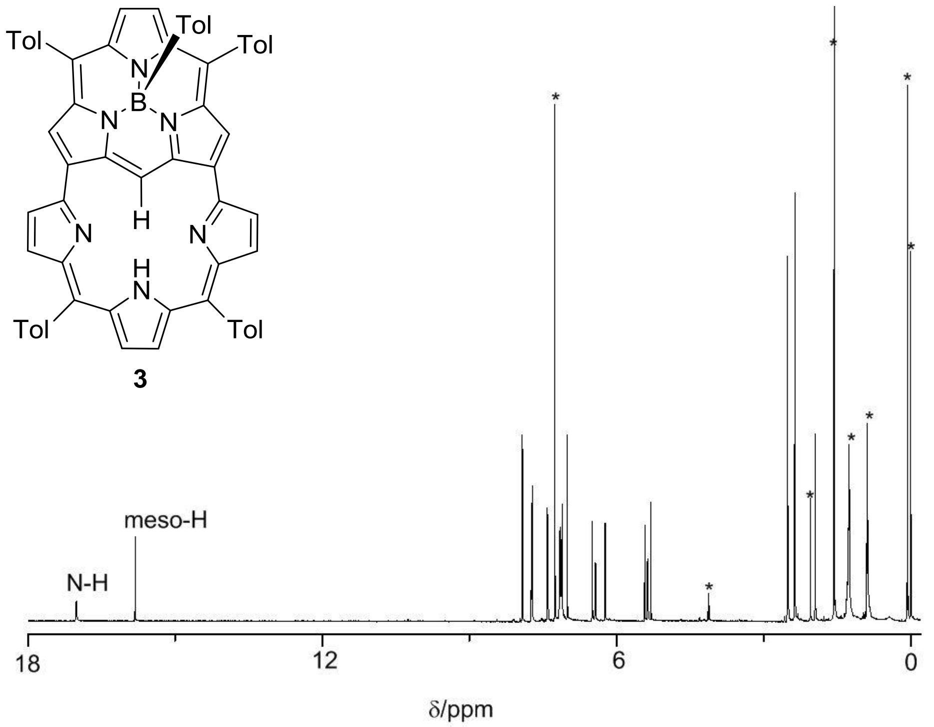

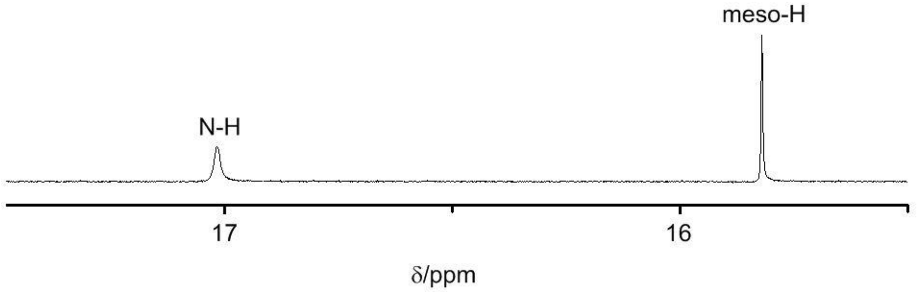

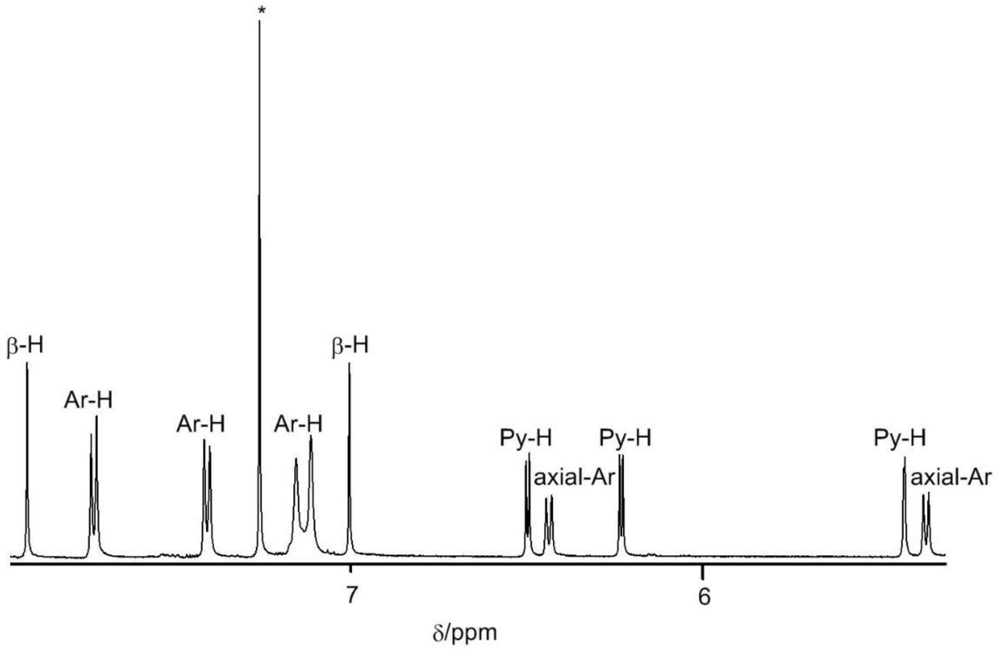

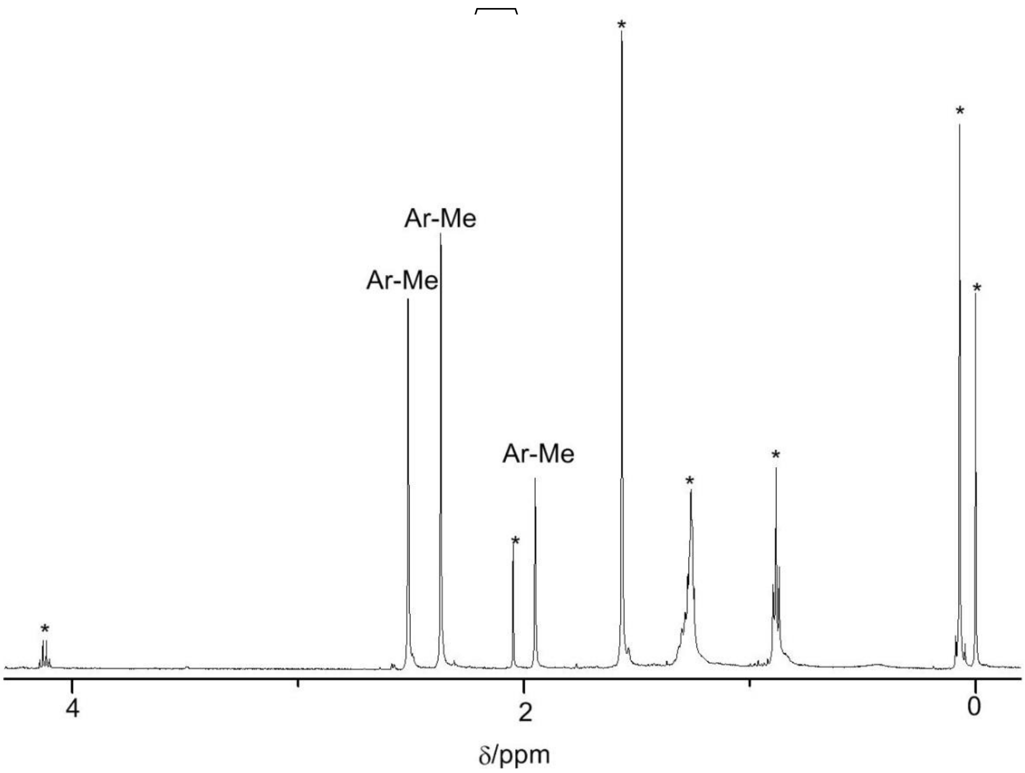

Figure S1: ^{1}H NMR spectrum of **3** in $CDCl_3$.

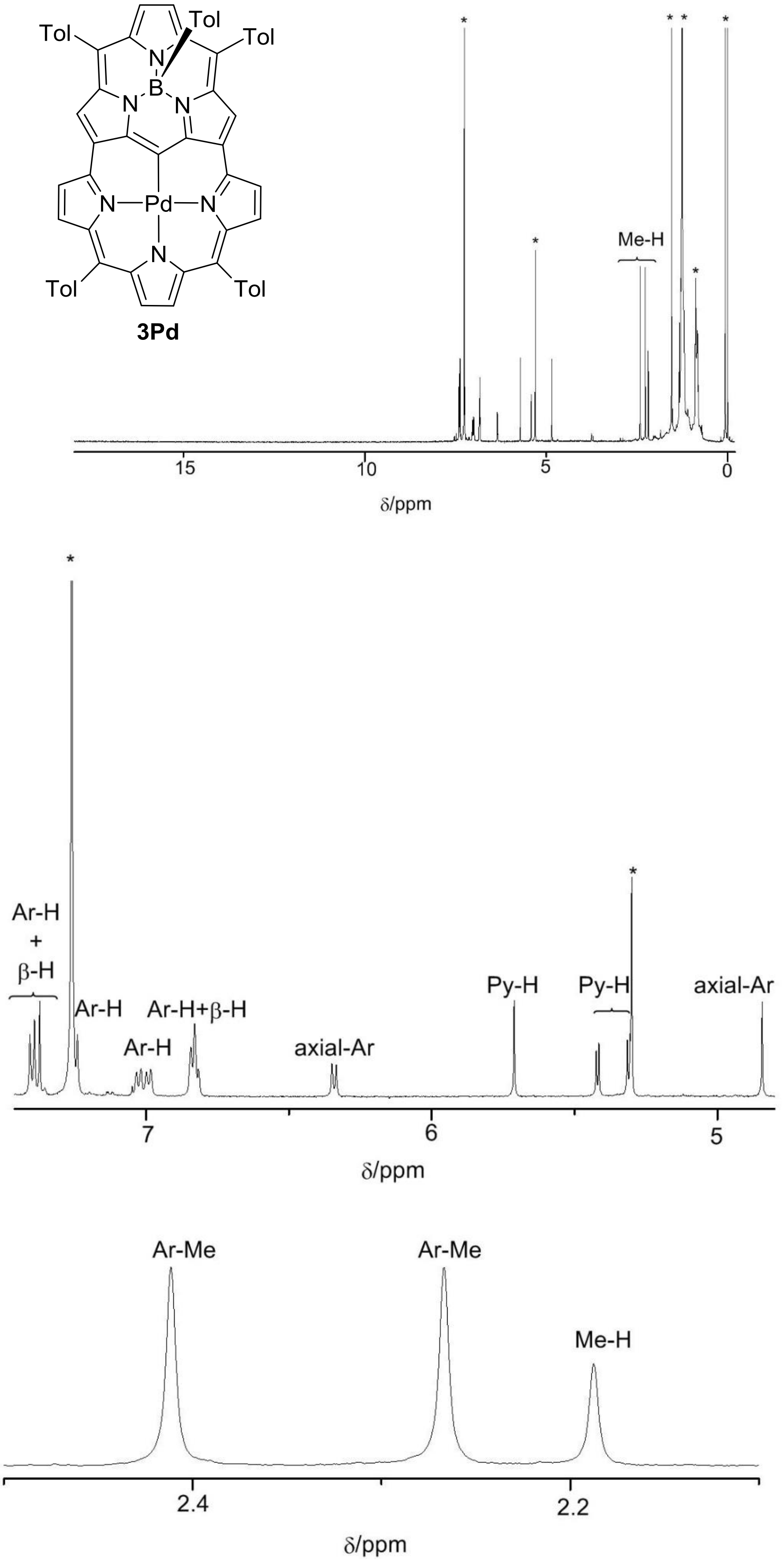

Figure S2: ^{1}H NMR spectrum of **3Pd** in $CDCl_3$.

X-ray crystal data

Table S1: Crystal data and structure refinement for **3**.

Empirical formula	$C_{64}H_{47}BN_6$	
Formula weight	910.88	
Temperature	100.02(10) K	
Wavelength	1.54184 Å	
Crystal system	Triclinic	
Space group	P-1	
Unit cell dimensions	a = 10.9229(3) Å	α= 81.9600(18)°.
	b = 14.2747(3) Å	β= 78.3826(18)°.
	c = 18.4446(4) Å	γ = 86.892(2)°.
Volume	2788.29(11) Å^3	
Z	2	
Density (calculated)	1.085 Mg/m^3	
Absorption coefficient	0.492 mm^{-1}	
F(000)	956	
Crystal size	0.2 x 0.15 x 0.02 mm^3	
Theta range for data collection	3.721 to 66.600°.	
Index ranges	-13<=h<=13, -16<=k<=17, -22<=l<=22	
Reflections collected	9813	
Independent reflections	9813 [R(int) = 0.0698]	
Completeness to theta = 66.600°	99.9 %	
Absorption correction	Semi-empirical from equivalents	
Max. and min. transmission	1.00000 and 0.61316	
Refinement method	Full-matrix least-squares on F^2	
Data / restraints / parameters	9813 / 0 / 645	
Goodness-of-fit on F^2	1.063	
Final R indices [I>2sigma(I)]	R1 = 0.0807, wR2 = 0.2472	
R indices (all data)	R1 = 0.1098, wR2 = 0.2562	
Extinction coefficient	n/a	
Largest diff. peak and hole	0.444 and -0.435 e.Å^{-3}	

a)

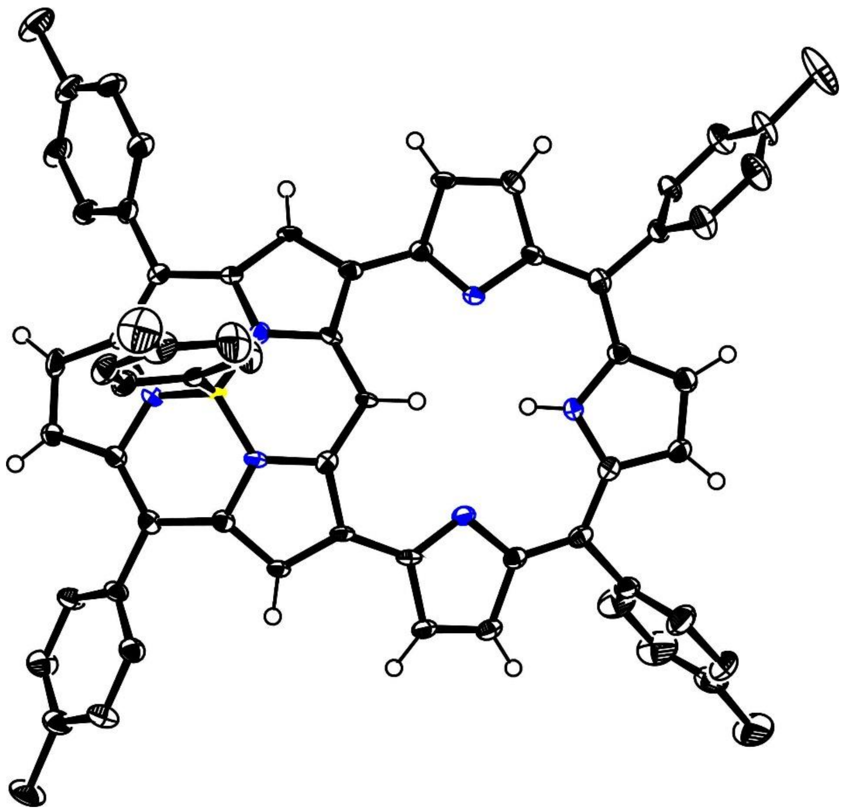

b)

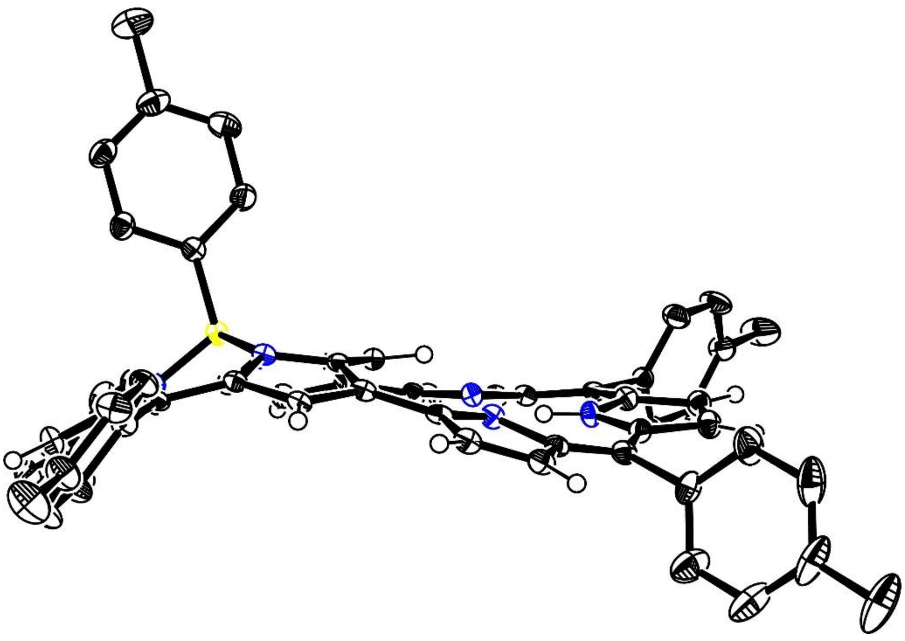

Figure S3: X-ray crystal structure of **3**. a) Top view, b) side view. The thermal ellipsoids are drawn at the 50% probability level. The contributions to the scattering arising from the presence of the disordered solvents in the crystal were removed by use of the utility SQUEEZE in the PLATON software package [S1].

Table S2: Crystal data and structure refinement for **3Pd**.

Empirical formula	$C_{64}H_{45}BN_6Pd$	
Formula weight	1015.27	
Temperature	100.01(10) K	
Wavelength	1.54184 Å	
Crystal system	Triclinic	
Space group	P-1	
Unit cell dimensions	a = 11.0064(3) Å	α= 82.748(2)°.
	b = 14.0560(3) Å	β= 78.001(2)°.
	c = 18.4924(5) Å	γ = 87.7004(19)°.
Volume	2775.71(12) Å^3	
Z	2	
Density (calculated)	1.215 Mg/m^3	
Absorption coefficient	3.031 mm^{-1}	
F(000)	1044	
Crystal size	0.2 x 0.1 x 0.02 mm^3	
Theta range for data collection	3.772 to 66.598°.	
Index ranges	-13<=h<=13, -17<=k<=17, -22<=l<=22	
Reflections collected	9791	
Independent reflections	9791 [R(int) = 0.0758]	
Completeness to theta = 66.598°	99.9 %	
Absorption correction	Semi-empirical from equivalents	
Max. and min. transmission	1.00000 and 0.56646	
Refinement method	Full-matrix least-squares on F^2	
Data / restraints / parameters	9791 / 0 / 654	
Goodness-of-fit on F^2	1.057	
Final R indices [I>2sigma(I)]	R1 = 0.0487, wR2 = 0.1262	
R indices (all data)	R1 = 0.0570, wR2 = 0.1303	
Extinction coefficient	n/a	
Largest diff. peak and hole	0.964 and -1.373 e.Å^{-3}	

a)

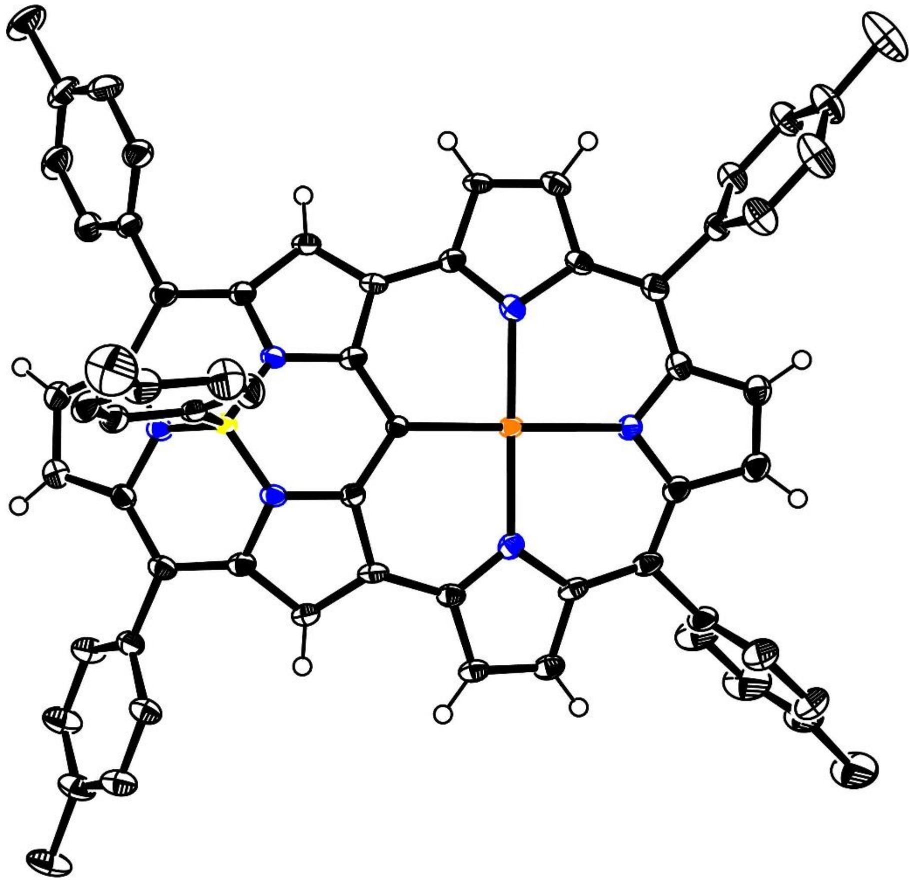

b)

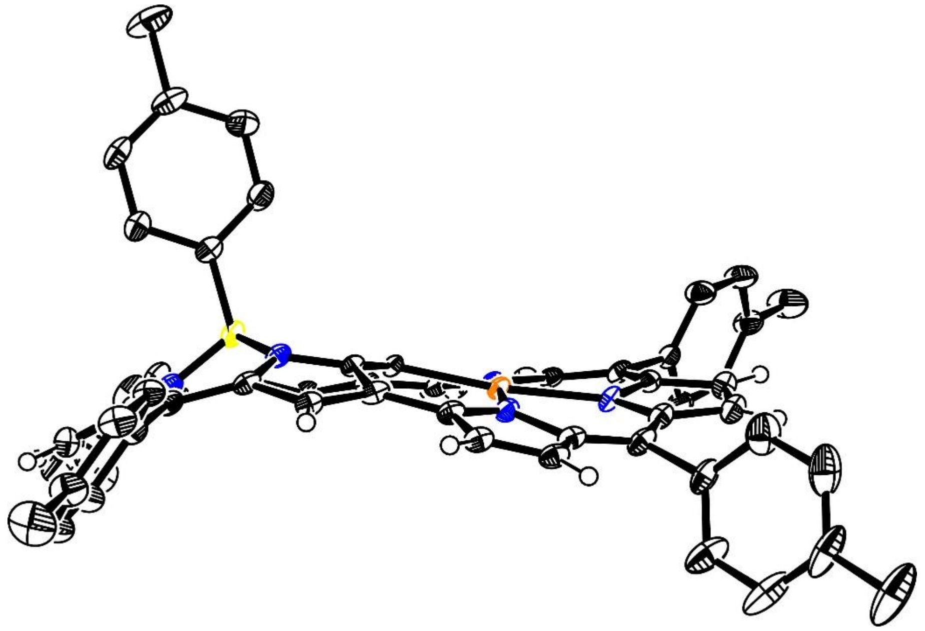

Figure S4: X-ray crystal structure of **3Pd**. a) Top view, b) side view. The thermal ellipsoids are drawn at the 50% probability level. The contributions to the scattering arising from the presence of the disordered solvents in the crystal were removed by use of the utility SQUEEZE in the PLATON software package [S1].

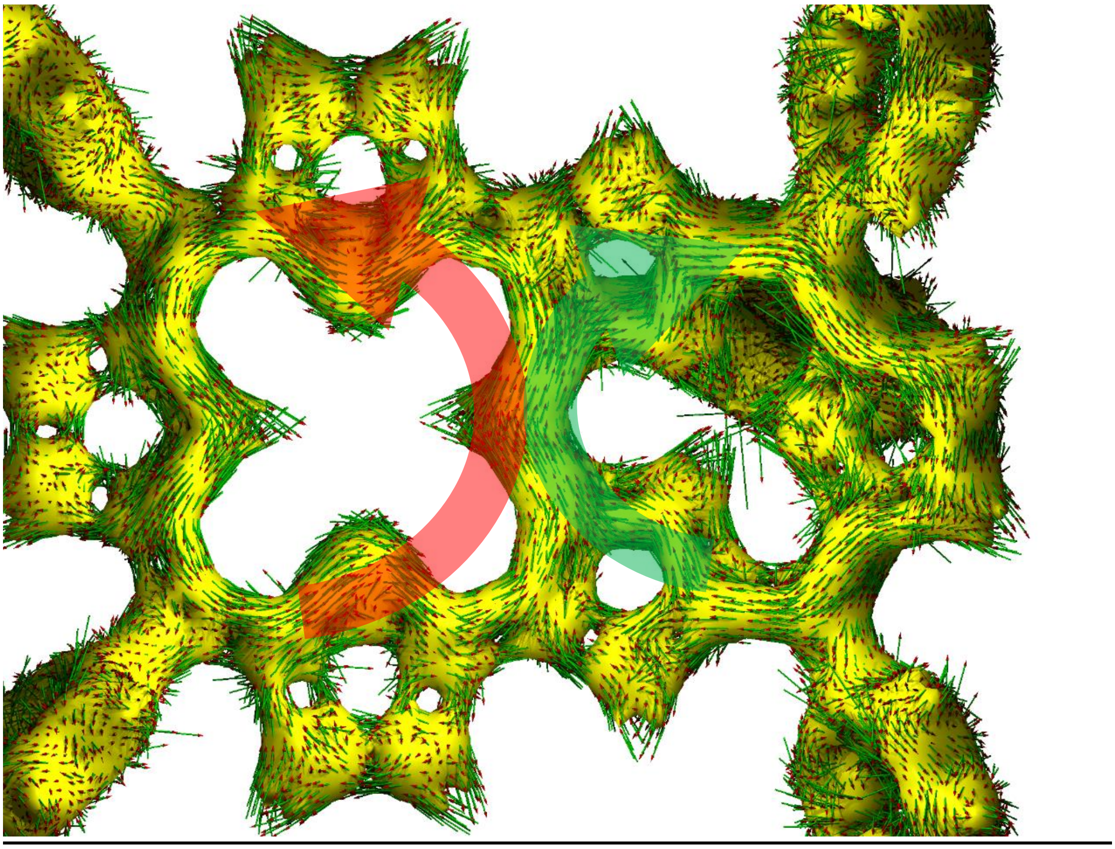

Figure S5: ACID diagram of **3.**

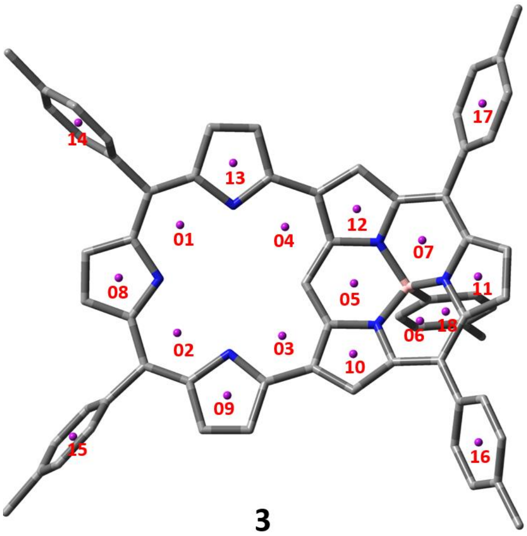

3

location	NICS(0)	NICS(1)	$NICS(1)_{zz}$	HOMA
01	11.2564	7.8298	28.3350	
02	10.8726	7.5429	27.7041	
03	12.6312	8.0890	32.2356	
04	12.7007	8.6245	32.0041	
05	-11.7037	-10.7424	-27.5577	
06	-9.6050	-9.4793	-23.1492	
07	-9.8633	-9.7895	-23.5719	
08	-4.5491	-4.1148	-9.4351	0.8953
09	-2.0790	-4.8661	-8.2386	0.2269
10	-1.5312	-3.2167	-0.4589	0.7093
11	-8.3236	-9.0218	-23.3478	0.6476
12	-1.5902	-3.3614	-2.0187	0.6969
13	-1.9697	-4.5945	-8.2000	0.2770
14	-8.1222	-9.6408	-26.7959	0.9906
15	-8.1976	-9.7591	-27.4141	0.9681
16	-6.9172	-8.7855	-24.5768	0.9743
17	-6.8786	-8.7608	-24.5577	0.9863
18	-9.1240	-11.2263	-26.6083	0.9875

Figure S6: NICS values of **3.**

References

[S1] Squeeze-Platon: a) Spek, A. L. PLATON, A Multipurpose Crystallographic Tool; Utrecht, The Netherlands, **2005**; b) van der Sluis, P. Spek, A. L. Acta Crystallogr. *Sect. A* **1990**, *46*, 194.
